# Pioglitazone modulates the proliferation and apoptosis of vascular smooth muscle cells via peroxisome proliferators-activated receptor-gamma

**DOI:** 10.1186/1758-5996-6-101

**Published:** 2014-09-19

**Authors:** Jing Wan, Zhichao Xiao, Shengping Chao, Shixi Xiong, Xuedong Gan, Xuguang Qiu, Chang Xu, Yexin Ma, Xin Tu

**Affiliations:** Department of Cardiology, Zhongnan Hospital of Wuhan University, Wuhan, Hubei China; Department of Cardiology, Tongji Medical College of Huazhong University of Science and Technology affiliated Tongji Hospital, Wuhan, Hubei China; Cardiovascular Research, Life Science and Technology College, Human Genome Research Center, Huazhong University of Science and Technology, Wuhan, Hubei China

**Keywords:** Peroxisome proliferators-activated receptor gamma, Thiazolidinedione, Apoptosis, Caspase, Cyclins

## Abstract

**Background:**

PPARγ is a member of the nuclear hormone receptor superfamily. It has been considered as a mediator regulating metabolism, anti-inflammation, and pro-proliferation in the Vascular Smooth Muscle Cells (VSMCs). Thiazolidinediones (TZDs), synthetic ligands of PPARγ, have anti-proliferative and pro-apoptotic effects on VSMCs, which prevent the formation and progression of atherosclerosis and restenosis following percutaneous coronary intervention (PCI). However, the underlying mechanism remains elusive. This present study therefore aimed to investigate the signaling pathway by which pioglitazone, one of TZDs, inhibits proliferation and induces apoptosis of VSMCs.

**Methods:**

The effects of pioglitazone on VSMC proliferation and apoptosis were studied. Cell proliferation was determined using BrdU incorporation assay. Cell apoptosis was monitored with Hoechst and Annexin V staining. The expression of caspases and cyclins was determined using real-time PCR and Western blot.

**Results:**

Pioglitazone treatment and PPARγ overexpression inhibited proliferation and induced apoptosis of VSMCs, whereas blocking by antagonist or silencing by siRNA of PPARγ significantly attenuated pioglitazone’s effect. Furthermore, pioglitazone treatment or PPARγ overexpression increased caspase 3 and caspase 9 expression, and decreased the expression of cyclin B1 and cyclin D1 in VSMCs.

**Conclusions:**

Pioglitazone inhibits VSMCs proliferation and promotes apoptosis of VSMCs through a PPARγ signaling pathway. Up-regulation of caspase 3 and down-regulation of cyclins mediates pioglitazone’s anti-proliferative and pro-apoptotic effects. Our results imply that pioglitazone prevents the VSMCs proliferation via modulation of caspase and cyclin signaling pathways in a PPARγ-dependent manner.

## Introduction

Proliferation and apoptosis of the Vascular Smooth Muscle Cells (VSMCs) play a key role in the development and progression of the atherosclerosis and restenosis after percutaneous coronary intervention (PCI) [1, 2]. Several signaling pathways are involved in the progression of atherosclerosis and restenosis and the key players include peroxisome proliferators-activated receptor gamma (PPARγ) [3], platelet-derived growth factors (PDGF) [4], endothelin-1 (ET-1) [5], thrombin, fibroblast growth factor (FGF) [6]. Activation and interplay of these molecules induce the proliferation and migration of VSMCs, leading to formation of artery plaque. Several drugs, such as pioglitazone, a synthetic ligand of PPARγ, have been developed to treat and prevent the proliferation of VSMCs by targeting individual factors of these pathways.

PPARγ is a member of the nuclear hormone receptor superfamily [7]. It has been initially considered as a mediator regulating glucose and lipid metabolism. More recently, studies have revealed the presence of PPARγ in endothelial cells (ECs), VSMCs, macrophages and cardiomyocytes. PPARγ has multiple functions, including anti-inflammation and pro-proliferation in VSMCs [8]. Apoptosis is an important contributor to the formation of atherosclerosis, especially in the process of restenosis after PCI. PPARγ has anti-apoptotic effect herein by modulation of caspase 3 [9].

Pioglitazone is commonly used as a primary anti-diabetic drug. Previous studies have shown that pioglitazone was able to inhibit the proliferation and induce apoptosis of VSMCs [10, 11]. However, the underlying mechanisms have not been well understood yet. Hence, the aim of present study was to determine if pioglitazone regulates cell cycle and caspase cascades, leading to an inhibition in VSMCs proliferation.

## Materials and methods

### Cell culture and in vitro cell treatment

Human coronary artery smooth muscle cells (Lonza, Basel Switzerland) were grown and maintained in SmGM-2 media (Lonza, Basel Switzerland) supplemented with 2% fetal calf serum, 10 ng/mL human epidermal growth factor, 1.0 mg/ml hydrocortisone, 12 mg/mL bovine brain extract, 50 mg/mL gentamicin, and 50 ng/mL amphotericin B at 37°C in 5% CO_2_ atmosphere. The purity of each VSMCs preparation in culture (>99%) was confirmed by immunocytochemistry for α-smooth muscle actin. VSMCs between passage 2 and 6 were used for following experiments. VSMCs (1 × 10^6^) were treated for 24 h in medium containing vehicle (0.5% methyl cellulose), 10 uM pioglitazone or 1 uM GW9662, (Sigma). To over-express PPARγ-1 in VSMCs, cells were transduced with the recombinant adenovirus at titers of 100 MOI for 24 hours. Wild type PPARγ-1 adenovirus (Ad-wt-PPARγ) and mutation PPARγ-1 adenovirus were kind gifts from Dr. Qinglin Yang (Morehouse School of Medicine, Atlanta, USA). According to the principles of siRNA design and the *PPARγ* gene sequence (GenBank Accession No. NM_005037), the duplexes of specific siRNA sequences 5′-GTTCAAACACATCACCCCC-3′ was synthesized, non targeting siRNA: 5′-GCATATTGTCTATGACCAACT-3′.

### Adenoviral transduction of VSMCs

The resultant recombinant virusmids were transfected into packaging cells HEK293 to generate recombinant adenoviruses. The primary crude lysates of the recombinant adenoviruses were prepared and purified by cesium chloride gradient ultracentrifugation as viral stocks and titrated using a standard plaque assay. VSMCs were seeded at a density of 2 × 10^5^ in one 6-cm dish in antibiotic-free medium containing 10% serum before incubation with the transduction reagent Oligofectamine (Invitrogen Carlsbad, CA). One day later, cells were transduced with the recombinant adenovirus at titers of 100 MOI for 24 hours following the manufacturer’s protocols. For co-transduction studies, attractene (Qiagen) was employed as the transduction agent following the manufacturer’s recommendations, and non-targeting siRNA was used as experimental controls. During the final 24 hours of transfection, cells were treated with either vehicle (0.5% methyl cellulose) or pioglitazone (1 μM) [12].

### BrdU cell proliferation assay

The cell proliferation assay was performed by measuring 5-Bromo-2′-deoxy-uridine (BrdU) (Roche Applied Science, USA) incorporation into the newly synthesized DNA of replicating cells. To determine cell proliferation, VSMCs were plated in 96-well plates and allowed to attach for 24 hours. Cells were then treated with 1 uM Pioglitazone, 10 uM GW9662 or transduced with the recombinant adenovirus for 24 hours. The cells were loaded with BrdU in the last 4 hours of treatment. BrdU incorporation was quantified by an immunofluorescence assay kit (Roche Applied Science, USA) following manufacturer’s instructions. Three fields were chosen randomly from various sections to ensure objectivity of sampling. Digital images were acquired using a confocal microscope. Each assay repeated three times. The total 100 cells from each field were counted, and BrdU positive cell and the ratio of BrdU positive cell versus 100 cells were calculated using a confocal microscope. Each assay repeated three times.

### Apoptosis of VSMCs detected by Hoechst staining

To evaluate morphologic changes of apoptotic VSMCs, morphology and apoptosis assay were performed using the Hoechst staining as described previously [9]. Briefly, cells were seeded on chamber slides, treated with 1 uM Pioglitazone, 10 uM GW9662 or transduced with the recombinant adenovirus for 24 h. Cells then were washed, fixed and stained with Hoechst 33258 (Sigma, St. Louis, MO). Dead cells and apoptotic bodies were identified by condensed or fragmented nuclei using a Nikon confocal microscope. The apoptotic scores were counted from five randomly selected fields by direct counting 500 cells in each sample using a blinded method [13]. The percentage of apoptotic cells was calculated as the number of apoptotic cells divided by the number of total cells.

### Measurement of apoptosis by flow cytometry

Apoptosis was measured using the FITC-Annexin V Apoptosis Detection kit (BD Bioscience, San Diego, CA) as described previously with modifications [14]. Briefly, VSMCs were harvested, incubated and treated with 10 uM GW9662, 1 uM pioglitazone or transduced with the recombinant adenovirus for 24 h. After cell treatment, VSMCs were washed twice with cold PBS and resuspended in 1× binding buffer, (10 mM HEPES/NaOH, pH 7.4, 140 mM NaCl, 2.5 mM CaCl2) at a concentration of 1 × 10^6^ cells/ml. Then 1 × 10^5^ cells in 100 μl binding buffer were transferred to 5 ml tubes and stained with 5 μl of FITC-Annexin V and 5 μl propidium iodide (PI). The cells were gently mixed and incubated at room temperature for 15 min. After washing the cells with 1× binding buffer to remove the excess FITC-Annexin V and PI, the cells were analyzed on a FACScan flow cytometer, which the wavelength of excitation and emission were 488 nm and 525 nm, respecrively. The data were analyzed using CellQuest software.

### Detection of active caspases 3/7, 8 and 9 in VSMCs

Caspases activities were measured using the Vybrant FAM caspase 3/7, 8 and 9 Assay Kit (Molecular Probes, Invitrogen) according to the manufacturer’s recommendations after the incubation of cells with pioglitazone (1uM), GW9662 (10uM), or transduced with the recombinant adenovirus for 24 h. The assay was performed on a fluorescent inhibitor of caspases (FLICA) methodology. The increase in the caspases activities was determined by comparing these results with the level of the untreated control. Samples analyzed on a FACScan flow cytometry with 488 nm excitation and green emission for the FLICA-stained cells.

### Western blot

After cell treatment, VSMCs were washed with phosphate-buffered saline (PBS) and lysed in RIPA buffer (Biotech, Shanghai, China). After one freeze/thaw cycle, lysates were centrifuged. Protein concentration was determined by a BCA protein assay (Biotech, Shanghai, China) using bovine serum albumin as the standard. A quantity amounting to 10 μg of protein sample was subjected to SDS-polyacrylamide gel electrophoresis. Proteins were then transferred to an ECL nitrocellulose membrane (Millipore). Incubating the membrane in Superblock (Pierce) for 1 h blocked nonspecific binding. Membranes were then incubated overnight at 4°C in primary antibodies, PPARγ1, Cyclin D1, Cyclin B1/cdc2 and β-actin (AbCam: ab8924, ab95281, ab7959, ab1801). All primary antibodies dilution was 1:1000 in each reaction. The blots were washed three times with TBST buffer and then incubated for 1 h at room temperature with anti-rabbit secondary antibody conjugated with horseradish peroxidase. Western blot analysis was conducted according to standard procedures using Supersignal chemiluminescence detection substrate (Pierce).

### Real time RT-PCR

Total RNA was extracted from VSMCs using TRIZOL reagent (Invitrogen, Carlsbad, CA, USA). cDNA was synthesized from 0.5 μg of total RNA with superscriptor reverse transcriptase (Invitrogen, Carlsbad, CA, USA). The following specific primers were used: PPARγ-forward: 5′-GCCCTTTACCACAGTTGATTTCTCCA-3′; PPARγ-reverse: 5′-TATCCCCACAGACTCGGCACTCA-3′; Cyclin B1/cdc2-forward: 5′-CTGGGTCGGGAAGTCACTGGAAAC-3′; Cyclin B1/cdc2-reverse: 5′-GCAGCATCTTCTTGGGCACACA-3′; Cyclin D1-forward: 5′-AGGCGGAGGAGAACAAACAGATCA-3′; Cyclin D1-reverse: 5′-AGAGGAAGCGTGTGAGGCGGTAGTA-3′; β-actin-forward: 5′-TTTTGTGCCTTGATAGTTCGC-3′; β-actin- reverse: 5′-GAGTCCTTCTGACCCATACCC-3′. The real-time PCR analysis was performed using SyBR-Green mix (Applied Biosystems, Carlsbad, CA) on a 7500 Real Time PCR station (Applied Biosystems). The results for real-time PCR were calculated as ratio target gene expression (experimental/ control) and were expressed as fold change.

### Statistical analysis

SPSS 11.0 software was used to for data analysis. Data were presented as mean ± SEM. Student’s t-test was employed to assess the statistical significance. *P* < 0.05 was regarded as significant.

## Results

### Manipulation of PPARγ expression in VSMCs

**Figure 1:**
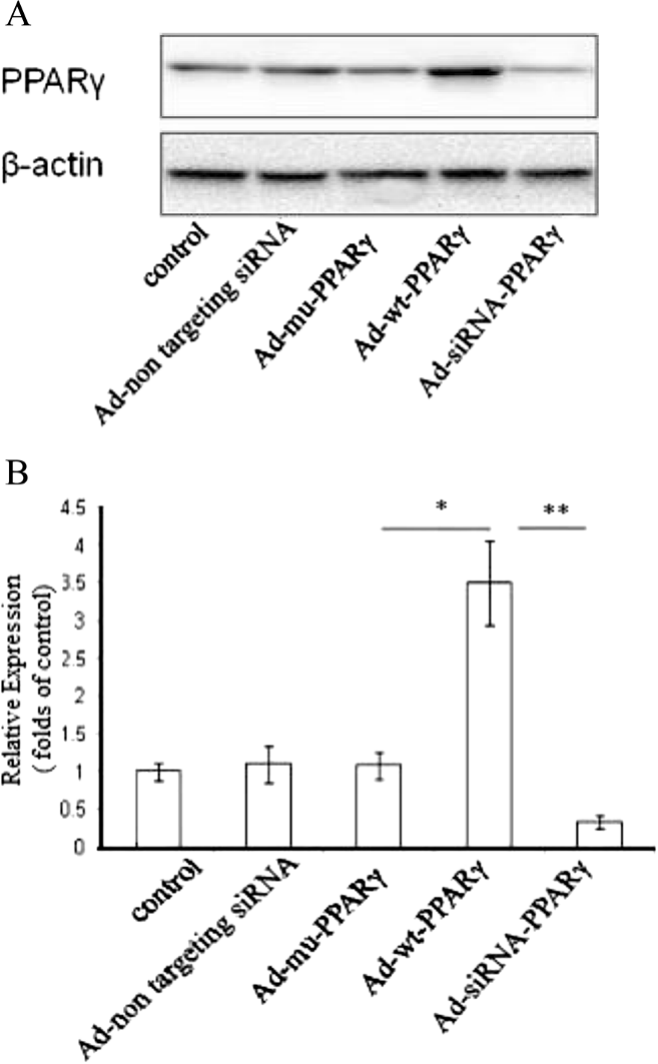
**Validation of overexpression and silencing of PPARγ. A**. Overexpression and silencing of PPARγ in VSMCs. VSMCs were transduced with Ad-mu-PPARγ, Ad-wt-PPARγ or Ad-siRNA-PPARγ, respectively. Cell pellets collected from different groups as indicated were probed against PPARγ and β-actin by Western blot. **B**. Quantification of PPARγ. The levels of PPARγ from distinct groups in (A) were quantified and normalized to the level of control. *P < 0.05, **P < 0.001. Each experiment has been repeated three times.

The expression levels of PPARγ in VSMCs were evaluated by Western blot after transduction with Adenovirus encoding wild type PPARγ (Ad-wt-PPARγ), mutant PPARγ (Ad-mu-PPARγ), or siRNA against PPARγ (Ad-siRNA-PPARγ). As shown in Figure 1, PPARγ protein levels were significantly increased in cells transduced with Ad-wt-PPARγ compared to Ad-mu-PPARγ (3.8 ± 0.57 vs. 1.09 ± 0.12, *P* < 0.05) (Figure 1B). On the other hand, silencing PPARγ in VSMCs has been achieved by Ad-siRNA-PPARγ comparing with Ad-wt-PPARγ was 0.34 ± 0.02 vs. 3.8 ± 0.57 (P < 0.001), and Ad-siRNA-PPARγ comparing with Ad non targeting siRNA was 0.147 ± 0.03 vs. 0.2415 ± 0.15 (P < 0.05) (Figure 1B). The efficiency of overexpression and knockdown of PPARγ has been determined.

### Pioglitazone inhibits VSMCs proliferation through PPARγ signaling pathway

**Figure 2:**
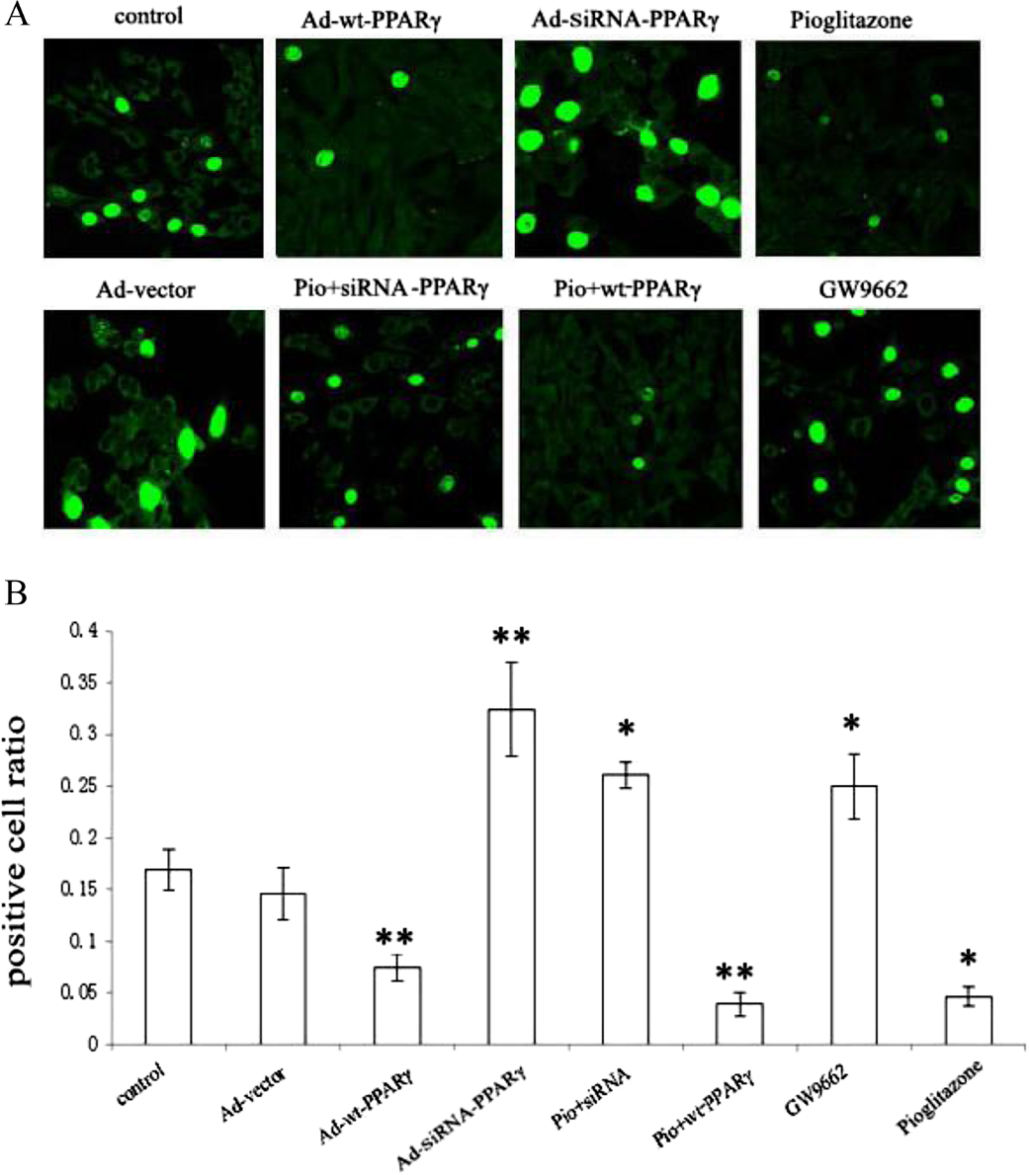
**Pioglitazone induced proliferation of VSMC through PPARγ. A**. Proliferation of VSMCs is regulated by pioglitazone through PPARγ signaling pathway. VSMCs alone (control), transduced with Ad-vector, Ad-wt-PPARγ, or siRNA-PPARγ were labeled with BrdU, respectively. In addition, VSMCs treated with pioglitazone (PIO) (1uM), wt-PPARγ-overexpressing VSMCs treated with PIO (Pio + wt-PPARγ), and PPARγ silenced VSMCs treated with PIO (Pio + siRNA) were harvested 24 h later after treatment followed by BrdU labeling. The BrdU positive VSMCs from indicated groups were recorded by confocal microscopy (600x). BrdU positive VSMCs cells from wt-PPARγ, PIO + wt-PPARγ, and PIO alone groups were less than those from control and vector alone groups. On the other hand, the addition of PIO in the absence of PPARγ (PIO + siRNA-PPARγ) and GW6992 alone induced the proliferation of VSMCs. The data is the representative of three individual experiments. **B**. The ratio of BrdU positive cells to total cells was quantified. The BrdU positive cell numbers and total cell numbers from each field were counted and the ratio of BrdU positive cell versus total cell numbers was calculated. **P < 0.01, *P < 0.05. Each experiment has been repeated three times.

We initially investigated whether pioglitazone inhibits proliferation of VSMCs. VSMCs were treated with pioglitazone (PIO), and the cell proliferation was determined by BrdU assay 24 hours later. As shown in Figure 2, a significant decrease in cell proliferation was observed in the PIO treated group compared with controls (0.051 ± 0.01 vs. 0.175 ± 0.031, *P* < 0.05).

We next determined if PPARγ pathway mediates pioglitazone’s anti-proliferative effect. VSMCs were treated with GW9662, a potent antagonist of PPARγ. GW9662 treatment significantly enhanced VSMCs proliferation (0.248 ± 0.054 vs. 0.175 ± 0.031, *P* < 0.05) (Figure 2). Furthermore, PPARγ silenced VSMCs were treated with pioglitazone. Interestingly, PPARγ silencing by siRNA in VSMCs totally abolished the inhibitory effects of PIO (0.279 ± 0.009 vs. 0.051 ± 0.01, *P* < 0.001) (Figure 2). Our results indicate that anti-proliferative effect of pioglitazone is mediated by PPARγ signaling pathway.

### Pioglitazone induced apoptosis in VSMCs

**Figure 3:**
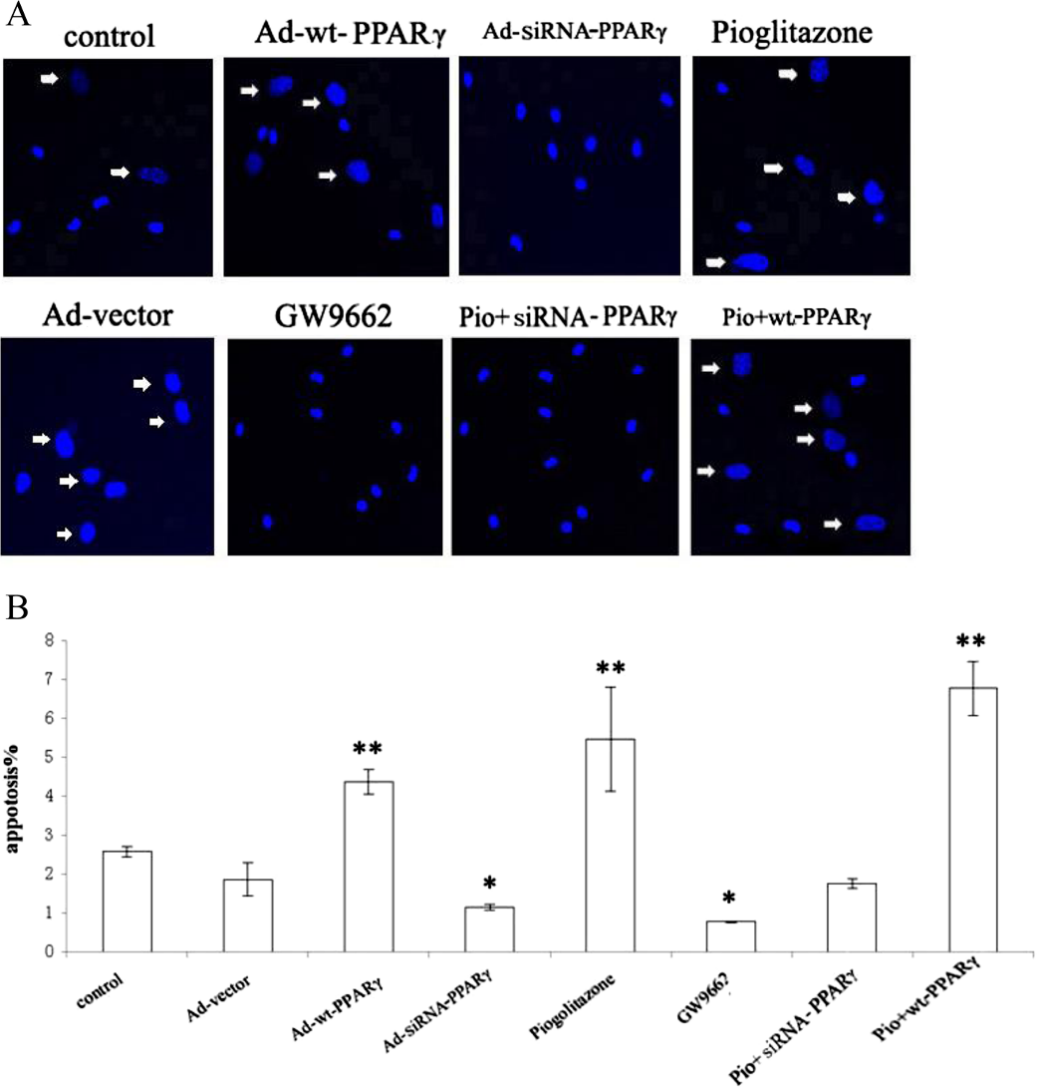
**Induction of chromatin condensation of VSMCs by piolitazone. A**. Pioglitazone induced chromatin condensation in the nuclei of VSMCs. VSMCs were seeded on top of coverslips and transfected with vector, wt-PPARγ, or siRNA-PPARγ, respectively. Cells (non-transduced and transduced) were then treated with different drugs as shown followed by Hoechst staining. Arrows indicate the nuclei with condensed chromatin. **B**. Quantification of condensed chromatin from apoptotic VSMCs. The numbers of VSMCs from different treatments were counted. The percentage of condensed-chromatin positive cells was determined. **P < 0.001, *P < 0.01. Values are presented as the mean ± SEM of three different fields. Each experiment has been repeated three times.

The viability of VSMCs was detected with chromatin condensation under the fluorescence microscope. Pioglitazone treatment or PPARγ overexpression significantly increased the numbers of condensed chromatin positive cells (5.51 ± 2.14% vs. 3.21 ± 0.27%, *P* < 0.01; and 6.32 ± 1.47% vs. 3.21 ± 0.27%, *P* < 0.001, respectively), indicating an increase in VSMC apoptosis by pioglitazone treatment. In contrast, GW9662 and PPARγ silencing significantly reduced cell numbers of undergoing apoptosis (1.67 ± 0.15% vs. 3.21 ± 0.27%, *P* < 0.01, and 1.17 ± 0.16% vs. 3.21 ± 0.27%, *P* < 0.001, respectively) (Figure 3).

**Figure 4:**
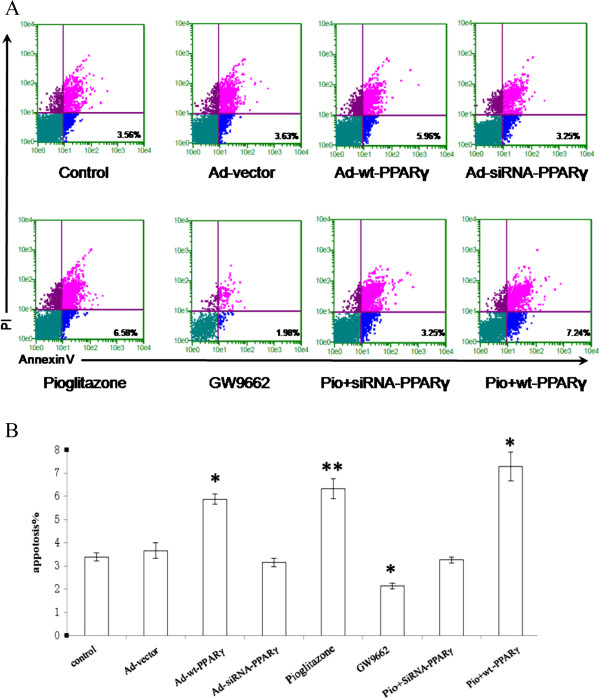
**Pioglitazone induced apoptosis of VSMCs. A**. Pioglitazone-treated VSMCs are inclined to apoptosis. Cultured VSMCs were transfected with vector, wt-PPARγ, or siRNA-PPARγ, respectively. Cells (non-transdued or transduced) were then treated with different drugs as indicated. VSMCs from distinct groups were then harvested and subjected to Annexin V staining. The representative staining plots **(A)** and the frequency of apoptotic cells from three independent experiments **(B)** was shown. The numbers indicate the frequency of the Annexin V+/PI- cell population. Values are presented as the mean ± SEM, n = 3. **P < 0.001, *P < 0.01.

The capability of pioglitazone to induce the apoptosis of VSMCs has been further confirmed using Annexin V staining (Figure 4). The frequency of the Annexin V^+^/PI^-^ cell population, which represents early apoptotic cell subset, has been indicated (Figure 4A). Pioglitazone increased apoptosis (6.38 ± 1.78% vs. 3.42 ± 0.16%, *P* < 0.001) and GW9662 reduced apoptosis (2.03 ± 0.11% vs. 3.42 ± 0.16%, *P* < 0.01). Overexpression of PPARγ was able to induce apoptosis of VSMCs (6.09 ± 0.12% vs. 3.58 ± 0.04%, *P* < 0.001). This effect was further enhanced by the treatment of pioglitazone (7.05 ± 0.24 vs. 3.58 ± 0.04%, *P* < 0.001). Silencing PPARγ completely blocked the induction of apoptosis mediated by Pioglitazone (Figure 4). These results suggest that pioglitazone induced VSMC apoptosis is in a PPARγ dependent pathway (Figure 4), which is in consistent with the results from BrdU staining (Figure 3).

### Pioglitazone treatment activates caspase3/7 and 8

**Figure 5:**
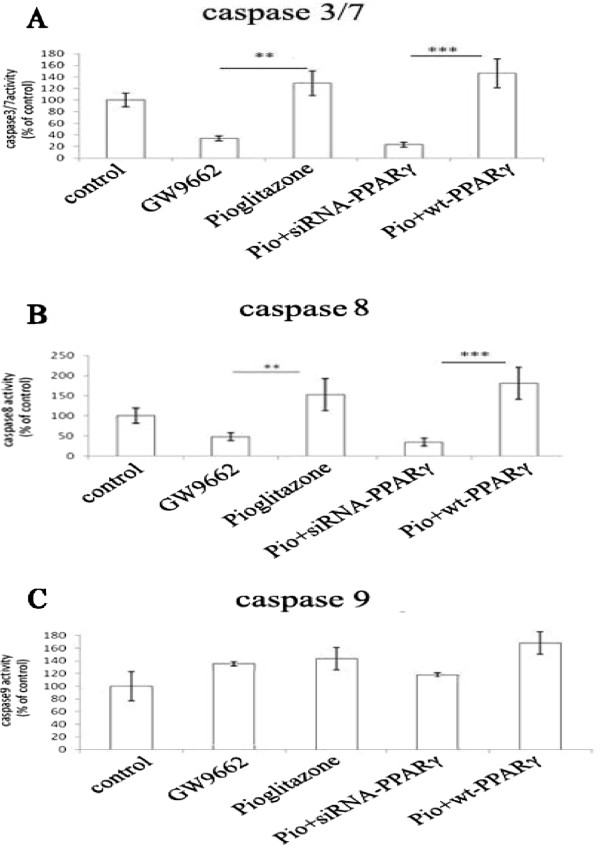
**Pioglitazone regulated activities of caspases. A**. The activity of the effector caspase3/7 was shown in the control, GW9662, Pioglitazone, PIO + siRNA-PPARγ and PIO + wt-PPARγ treated VSMCs, respectively. **B**. The activity of the initial caspase of intrinsic pathway-caspase8 in different gr006Fups. **C**. The activity of the initial caspase of extrinsic pathway-caspase9 in different groups. **P < 0.001, ***P < 0.0001. The activity of different caspases is normalized to the control (100%). Percentage is presented as the mean ± SEM of triplicates. Each experiment has been repeated three times.

Activated caspase 3/7 is a critical effector in the apoptosis signal pathway. Caspase 8 is a factor related to the extrinsic pathway, and caspase 9 is a key member along with intrinsic pathway. To further investigate whether activated caspases were involved in anti-proliferative effect of pioglitazone, activities of caspase3/7, 8, 9 were measured in PIO treated VSMCs. As shown in Figure 5, PIO treatment resulted in significant increases in activated caspase 3/7 (121.67 ± 3.06%) and caspase 8 (151.2 ± 7.03%), but not in activated caspase 9. A similar effect was seen when wt-PPARγ was overexpressed in VSMCs (137.76 ± 1.91%, 173.56 ± 13.12%). In contrast, GW9662 treatment resulted in significant decreases in the activation of caspase 3/7 (20.67 ± 2.51%) and caspase 8 (42.27 ± 1.46%) (Figure 5). Silencing PPARγ inhibited pioglitazone’s effect on the activation of caspase 3/7 (10.67 ± 1.53%) and caspase 8 (24.67 ± 3.1%), but has no influence on caspase 9 activation (Figure 5). These results suggest that pioglitazone activates the extrinsic, not the intrinsic pathway through a PPARγ dependent pathway.

### Effect of pioglitazone on cyclins in VSMCs

**Figure 6:**
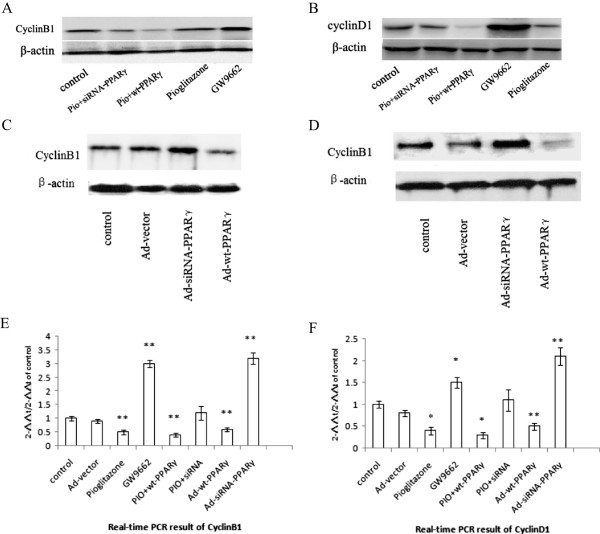
**Pioglitazone down-regulated the expression levels of the cyclin B1 and cyclin D1 in VSMCs. A**, **B**, The protein levels of cyclin B1 and cyclin D1 from VSMCs treated with GW9662, Pioglitazone, PIO + wt-PPARγ, or PIO + siRNA-PPARγ were analyzed by Western blot, respectively. **C**, **D**. The protein levels of cyclin B1 and cyclin D1 from VSMCs transduced with control, Ad-wt-PPARγ or Ad-siRNA-PPARγ were shown. One representative blot is shown. Each experiment has been repeated at least three times. **E** and **F**, the mRNA levels of cyclin B1 and cyclin D1 in VSMCs treated with GW9662, Pioglitazone, PIO + wt-PPARγ, or PIO + siRNA-PPARγ were analyzed by real-time RT-PCR. The transcripts of cyclin B1 and cyclin D1 were also determined in PPARγ-overexpressed or -silenced VSMCs. Values are presented as the mean ± SEM. *P < 0.01, **P < 0.001. Each experiment has been repeated three times.

To address the possibility that the down-regulation of cyclins in pioglitazone treated VSMCs resulted in the inhibition of cell proliferation, the effect of pioglitazone on transcription and expression levels of cyclin B1 and D1 were examined by RT-PCR and Western blot. As shown in Figure 6, pioglitazone treatment significantly down-regulated expression of cyclin B1 and cyclin D1 at both mRNA (0.46 ± 0.08, 0.41 ± 0.11) and protein (0.75 ± 0.12, 0.54 ± 0.17) levels. In contrast, GW9662 up-regulated the mRNA (2.73 ± 0.22, 1.49 ± 0.31) and protein (2.18 ± 0.25, 3.32 ± 0.72) levels of cyclin B1and cyclin D1. Moreover, PPARγ silencing completely abolished pioglitazone’s effect. These results suggest that cyclin pathway may involve in anti-proliferative effect of pioglitazone.

## Discussion

The restenosis after PCI has become one of the most concerned issues worldwide [15]. The proliferation and apoptosis of VSMCs play critical roles in this pathologic process [16]. Thus, many therapeutic treatments focused on preventing VSMC proliferation and inducing apoptosis in VSMCs. TZD is one of the most well studied agents. TZDs are synthetic ligands of PPARγ, which is a member of the nuclear hormone receptor super-family. TZDs are primarily used as anti-diabetes drugs. TZD has also been indicated to have an anti-proliferative function in rat renal arteriolar smooth muscle cells [17]. Recently, given their anti-proliferative and pro-apoptotic effect, TZDs have been considered as novel drugs to prevent or even reverse the formation of atherosclerosis and post-PCI restenosis. Importantly, TZDs do not increase the risk of overall cardiovascular morbidity or mortality in comparison with standard glucose-lowering drugs [18, 19], although there was the controversy that TZDs might potentially lead to serious adverse cardiovascular effects, such as heart failure after treatment with rosiglitazone for type 2 diabetes [20–22] . However, the mechanisms by which TZDs regulate VSMC proliferation have not been determined.

Previous studies have shown that TZDs suppress the expression of inflammatory molecules, including TNF (tumor necrosis factor)-α, MCP (monocyte chemotactic protein)-1, IL-1β and IL-6 in VSMCs [23, 24]. Moreover, recent studies have revealed that c-fos was involved in PPARγ agonists- induced growth suppression in VSMCs, and TZDs inhibited the expression of c-fos via the blockade of MAPK pathway [25]. Furthermore, Eukaryotic initiation factor 4E-binding protein (4EBP) and Src homology 2–containing inositol phosphatase 2 (SHIP2) mediate the inhibitory effects of TZD on cell growth [26]. Finally, it has been shown that TZDs prevent G1/S phase transition in PDGF or insulin stimulated VSMCs, suggesting that TZDs can induce cell cycle arrest [27].

It has been known that TZDs have anti-proliferative effect in different cell types via PI3-Kinase pathway [28]. However, whether this effect is PPARγ-dependent remains to be clarified [29, 30]. The effects of PPARγ in the vascular cells indicate its beneficial function in vascular disorders including hypertension and atherosclerosis [31]. Goetze group [29] has shown that troglitazone inhibited insulin-induced mitogenic signaling through a PPARγ-mediated inhibition of ERK-dependent phosphorylation and activation of nuclear transcription factors. However, this group revealed that TZDs activated MEK/ERK pathway through PI3-kinase and promoted c-fos mRNA expression and DNA synthesis, a process independent of PPARγ pathway [30]. Cersosimo group suggested pioglitazone preserved Akt phosphorylation and attenuates MAPK signaling in insulin-stimulated VSMCs, and may play a role in arterial smooth muscle cells migration, proliferation, and inflammation in conditions of acute hyperinsulinemia [32]. In our study, we found that pioglitazone treatment and PPARγ overexpression inhibited VSMC proliferation. Whereas silencing PPARγ with siRNA attenuated the inhibitory effects. These results clearly indicate that the PPARγ signaling pathway is involved in anti-proliferative effect of pioglitazone. Pioglitazone is already shown to inhibit in-stent neointimal formation in humans [33].

Cyclins play critical roles in cell cycle regulation, especially cyclin B1 and cyclin D1 [34]. PPARγ ligands inhibited G_1_ to S transition by inhibiting the expression of minichromosome maintenance (MCM) gene, one of the downstream effector factors of pRB/E2F pathway [35]. Stimulation of PPARγ induced the arrest of cell cycle, accompanied by the down-regulation of cyclin D and cyclin B in VSMCs [36–39]. To determine the detailed mechanisms by which pioglitazone regulates VSMC proliferation, in this study the mRNA and protein levels of cyclin B1 and cyclin D1 were tested. We found that pioglitazone treatment and PAPRγ overexpression significantly down-regulated both mRNA and protein levels of cyclin B1 and cyclin D1. These results suggest that the beneficial functions of the TZDs are mediated, at least in part, by regulating the expression and transcription of cyclin B and cyclin D.

Aside from the impact of proliferation of VSMCs on the formation of atherosclerosis and restenosis, the apoptosis of VSMC also plays an important role in these processes. The pro-apoptotic effect of PPARγ in VSMCs has been reported. Bruemmer’s group revealed that the Oct-1 protein was regulated by the TZDs, which in turn induced overexpression of the growth arrest and DNA damage inducible protein 45(GADD45) gene, ultimately leading to the apoptosis of VSMCs [40]. Other groups have shown that pioglitazone also activated TGF (Transforming Growth Factor)-β-smad2-GADD45 pathway [41, 42]. Pioglitazone induced apoptosis in VSMCs through Smad2 phosphorylation [11]. Caspases are a family of cysteine proteases that play important roles in apoptosis. Caspase 8 and caspase 9 are the two initiative caspases involved in both extrinsic and intrinsic apoptotic pathways, while caspase 3 is a terminal effector caspase [43]. Bruedigam group showed that rosiglitazone stimulated mineralization by induction of caspase-dependent apoptosis [44]. Here, we found that pioglitazone treatment and PPARγ overexpression induced activation of caspase 8 and caspase 3/7, indicating that pioglitazone induces VSMC apoptosis through the extrinsic caspase pathway.

In summary, our study shows for the first time the regulatory pathways involved in the anti-proliferative effect of pioglitazone in VSMCs. Pioglitazone treatment inhibits proliferation of the VSMCs and induces VSMC apoptosis in a PPARγ-dependent pathway. Down-regulation of cyclin B1 and cyclin D1 and activation of caspase 8 and caspase 3/7 may be one of the mechanisms by which pioglitazone inhibits VSMC proliferation.

## Abbreviations

TZDs: Thiazolidinediones
PPARγ: Peroxisome proliferators-activated receptor gamma
VSMCs: Vascular smooth muscle cells
PCI: Percutaneous coronary intervention
CAD: Coronary artery disease
PDGF: Platelet-derived growth factors
ET: Endothelin
FGF: Fibroblast growth factor
IL: Interleukin
ECs: Endothelial cells
TNF: Tumor necrosis factor
MCP: Monocyte chemotactic protein
EBP: Eukaryotic initiation factor 4E-binding protein
SHIP2: Src homology 2–containing inositol phosphatase 2
GADD: Growth Arrest and DNA Damage
TGF: Transforming growth factor
PIO: Pioglitazone
